# Epidemiology of Pediatric Traumatic Brain Injury and Hypothalamic-Pituitary Disorders in Arizona

**DOI:** 10.3389/fneur.2019.01410

**Published:** 2020-01-22

**Authors:** J. Bryce Ortiz, Alona Sukhina, Baran Balkan, Gevork Harootunian, P. David Adelson, Kara S. Lewis, Oliver Oatman, Vignesh Subbian, Rachel K. Rowe, Jonathan Lifshitz

**Affiliations:** ^1^Translational Neurotrauma Research Program, Department of Child Health, University of Arizona College of Medicine-Phoenix, Phoenix, AZ, United States; ^2^Barrow Neurological Institute at Phoenix Children's Hospital, Phoenix, AZ, United States; ^3^College of Engineering, University of Arizona, Tucson, AZ, United States; ^4^Center for Health Information and Research, Arizona State University, Tempe, AZ, United States; ^5^Endocrinology, Phoenix Children's Hospital, Phoenix, AZ, United States; ^6^Phoenix Veteran Affairs Health Care System, Phoenix, AZ, United States

**Keywords:** traumatic brain injury, pediatrics, endocrine dysfunction, concussion, adolescence, hypopituitarism, puberty, head injury

## Abstract

Traumatic brain injury (TBI) in children can result in long-lasting social, cognitive, and neurological impairments. In adults, TBI can lead to endocrinopathies (endocrine system disorders), but this is infrequently reported in children. Untreated endocrinopathies can elevate risks of subsequent health issues, such that early detection in pediatric TBI survivors can initiate clinical interventions. To understand the risk of endocrinopathies following pediatric TBI, we identified patients who had experienced a TBI and subsequently developed a new-onset hypothalamic regulated endocrinopathy (*n* = 498). We hypothesized that pediatric patients who were diagnosed with a TBI were at higher risk of being diagnosed with a central endocrinopathy than those without a prior diagnosis of TBI. In our epidemiological assessment, we identified pediatric patients enrolled in the Arizona Health Care Cost Containment System (AHCCCS) from 2008 to 2014 who were diagnosed with one of 330 TBI International Classification of Diseases (ICD)-9 codes and subsequently diagnosed with one of 14 central endocrinopathy ICD-9 codes. Additionally, the ICD-9 code data from over 600,000 Arizona pediatric patients afforded an estimate of the incidence, prevalence, relative risk, odds ratio, and number needed to harm, regarding the development of a central endocrinopathy after sustaining a TBI in Arizona Medicaid pediatric patients. Children with a TBI diagnosis had 3.22 times the risk of a subsequent central endocrine diagnosis compared with the general population (±0.28). Pediatric AHCCCS patients with a central endocrine diagnosis had 3.2-fold higher odds of a history of a TBI diagnosis than those without an endocrine diagnosis (±0.29). Furthermore, the number of patients with a TBI diagnosis for one patient to receive a diagnosis of a central endocrine diagnosis was 151.2 (±6.12). Female subjects were more likely to present with a central endocrine diagnosis after a TBI diagnosis compared to male subjects (64.1 vs. 35.9%). These results are the first state-wide epidemiological study conducted to determine the risk of developing a hypothalamic-pituitary disorder after a TBI in the pediatric population. Our results contribute to a body of knowledge demonstrating a TBI etiology for idiopathic endocrine disorders, and thus advise physicians with regard to TBI follow-up care that includes preventive screening for endocrine disorders.

## Introduction

In children, traumatic brain injuries (TBIs) account for over 812,000 emergency department visits every year and are a leading cause of childhood mortality and morbidity in the United States (1). A TBI can be defined as a non-degenerative, non-congenital insult to the brain from an external mechanical force, potentially leading to permanent or temporary impairment of cognitive, physical, and psychosocial functions, with an associated diminished or altered state of consciousness. Those who survive pediatric TBI are at risk for poor developmental and functional outcomes later in life. Very young children may be particularly vulnerable to the effects of TBI as the brain is under continuous development throughout childhood (2). Pediatric survivors of TBI are at increased risk for worse behavioral, social, and academic outcomes compared to their peers (3–6). Moreover, pediatric survivors of TBI show high incidence of health issues including pain, cardiovascular, and metabolic disorders (2). In particular, TBI precede the development of endocrinopathies, or dysfunction of the endocrine system, as reported in adults (7–11). Furthermore, both early and late endocrine changes can occur after TBI in pediatric patients (12). These alterations include acute alterations in the hypothalamic-pituitary-adrenal axis, antidiuretic hormone regulation, growth hormone (GH) deficiency, disturbances in puberty, central hypothyroidism [hypothyroidism due to insufficient stimulations by thyroid stimulating hormone (TSH) of an otherwise normal thyroid gland], and elevated prolactin, which can each be temporary or permanent (12, 13). The goal of this study was to better understand the epidemiology of TBI and subsequent endocrinopathy in the pediatric population. We hypothesized that pediatric patients who were diagnosed with a TBI were at greater risk of being diagnosed with a hypothalamic-pituitary disorder than those without a prior diagnosis of TBI.

In adults, the prevalence of endocrinopathy following TBI is common, with the most prevalent disorder being GH deficiency (7, 10, 14, 15). A recent meta-analysis that included data from 2,756 adult TBI patients reported a 32% overall prevalence of at least one endocrine diagnosis after TBI (8). Similarly, a previous meta-analysis that included data of 1,203 adult TBI patients, reported a 27.8% overall prevalence of at least one endocrine dysfunction, with 6.2% of patients having more than one endocrine dysfunction post-TBI (16). However, the prevalence of endocrine dysfunction after TBI in the pediatric population is less clear. Between 1977 and 2004, only a total of 20 pediatric cases of hypopituitarism after TBI were reported. Across these reports, the interval between TBI and endocrine diagnosis ranged from 1 to 42 years (17), which highlights the lack of recorded data in this domain. The prevalence of endocrine dysfunction following a TBI in pediatric studies ranged from 5 to 57% and up to 86% in studies including hyperprolactinemia as an abnormality (17). However, more recent studies show that endocrine dysfunction may be a common occurrence following pediatric TBI (18–23). These existing reports differ in the eligible pediatric population, inclusion criteria, and methodological design, in addition to varied hormonal assessment, baseline profile, and dynamic tests, sometimes in subsets of subjects (24–30), which compromise the ability to compare between studies. Thus, there is a need to better understand whether a TBI diagnosis is a risk factor for a subsequent endocrine diagnosis in the pediatric population. Here, we present the epidemiology of central endocrine diagnoses following a patient's first TBI diagnosis in the Arizona pediatric population, with a focus on male/female patients and time between TBI and endocrine diagnoses.

## Methods

### Inclusion and Exclusion Criteria

We used de-identified patient records from the Arizona Health Care Cost Containment System (AHCCCS), the Medicaid program for the state of Arizona. We queried the AHCCCS database for patients (≤ 18 years old) with a TBI diagnosis followed by a central endocrine diagnosis after the initial TBI diagnosis (see Appendices 1, 2 for International Classification of Diseases (ICD)-9 diagnoses codes for TBI and endocrinopathies, respectively). Based on clinical relevance and inputs from care providers, TBI diagnoses were restricted to a total of 330 diagnoses of concussion, skull fracture, cerebral injury or hemorrhage, and head injury; cerebrovascular diseases were excluded. Central endocrine diagnoses were restricted to 14 diagnoses of the pituitary, the hypothalamus, diabetes insipidus, and puberty, excluding premorbid diabetes, toxic exposure, and circadian rhythm disorders. Inclusion/exclusion criteria for the research study primarily identified cases of TBI followed by an endocrine diagnosis, with both diagnoses having occurred before or at the age of 18. In addition, we included only patients who were continuously enrolled in AHCCCS with no more than a 30-day gap in coverage per year, in order to assure that missed diagnoses were minimized. The number of patients found in these records was sufficient to conduct an epidemiological study to determine the relationships between age and gender (only male and female) of patients with a TBI diagnosis and subsequent endocrine diagnosis. The inclusion/exclusion criteria for the population is presented in Table 1. The study protocol was reviewed and approved by the Phoenix Children's Hospital Institutional Review Board (IRB 15-021) and deferred by the University of Arizona and Arizona State University.

**Table 1:** Inclusion/exclusion criteria for the sample population.

**Inclusion criteria**
Age 0 ≤ 18 years
Located in Arizona
Enrolled in AHCCCS Medicaid from 2008 to 2014 without a lapse >30 days
Diagnosis of TBI
Diagnosis of an endocrine disorder after the TBI diagnosis
All diagnoses prior to or at the age of 18
Patients diagnosed with a TBI prior to or at the age of 18, without an endocrine disorder
Patients diagnosed with an endocrine disorder prior to or at the age of 18, without a TBI
**Exclusion criteria**
Diagnosis of an endocrine disorder prior to TBI

*AHCCCS, Arizona Health Care Cost Containment Service; TBI, Traumatic Brain Injury*.

### Data Analysis

To evaluate the risk of endocrine diagnosis after TBI diagnosis, a limited data set with ICD-9 diagnosis and billing codes was extracted for individual patients in the sample. Demographic data and care delivery dates populated four cohorts (see contingency matrix in Table 2): patients diagnosed with TBI and subsequent endocrine disorder (TBI+, Endo+; A), with TBI and without endocrine disorder (TBI+, Endo–; B), without TBI and with endocrine disorder (TBI–, Endo+; C), and with neither TBI nor endocrine diagnoses (TBI–, Endo–; D). Based on the contingency table and demographic data, calculations produced the prevalence, incidence, relative risk, odds ratio, attributable risk, and number needed to harm of endocrine diagnosis after TBI diagnosis stratified by age and male/female patients. Data pre-processing and analyses were performed using Python (version 3.7.3). Prevalence was calculated as the number of TBI patients with an endocrine diagnosis divided by those TBI patients without an endocrine diagnosis for each year (*A*/*C*). Incidence rate was calculated as the number of new cases per year of endocrine diagnoses in TBI patients divided by the cumulative population of TBI patients (*A*/(*A* + *B*)). Relative risk was calculated as the ratio of endocrine diagnoses in subjects with and without TBI $\left(\frac{A}{\left(A+B\right)}/\frac{C}{\left(C+D\right)}\right)$. Odds ratio was calculated as the ratio of patients with endocrine diagnoses and TBI to those without TBI as a fraction of patients without endocrine diagnoses and TBI to those without TBI $\left(\frac{A}{C}/\frac{B}{D}\right)$. Attributable risk was calculated as the difference in risk between patients with TBI and those without TBI $\left(\frac{A}{A+B}-\frac{C}{C+D}\right)$. Number needed to harm was calculated as the inverse of the attributable risk $\left(\frac{1}{Attributable\;Risk}\right)$. Epidemiological calculations were performed for each year, averaged over the 7 years. Prevalence of TBI and endocrine dysfunction are reported as mean and 90% confidence interval.

**Table 2:** Contingency table of sample populations for 2008–2014.

	**TBI+**		**TBI–**	
**Year**	**Endo+**	**Endo–**	**Endo+**	**Endo–**
2008	144	15,238	1,334	568,645
2009	154	16,919	1,585	646,008
2010	191	20,389	1,945	606,738
2011	220	20,906	2,024	622,756
2012	221	22,597	2,168	629,391
2013	223	22,153	2,237	616,052
2014	232	22,353	2,137	657,512

*Contingency table of the sample populations used in this study. The table shows the database population for each year included in the analysis. TBI+, traumatic brain injury diagnosis present; TBI–, TBI diagnosis absent; Endo+, central endocrine diagnosis present; Endo–, central endocrine diagnosis absent*.

## Results

### Prevalence of TBI and Endocrine Dysfunction Stratified by Age

The AHCCCS provided care for an average of 643,212 (±19,097 at CI of 90%) pediatric patients per year for analysis. From the 275,781 unique patients with either a TBI or central endocrine diagnosis, there were 498 unique patients who were diagnosed with a TBI and a subsequent endocrine diagnosis between 2008 and 2014, with an annual average of 197.9 (±22.3 at CI of 90%) unique patients within each year. A total of 107,458 children were diagnosed with a TBI before or at the age of 18 years.

**Figure 1:**
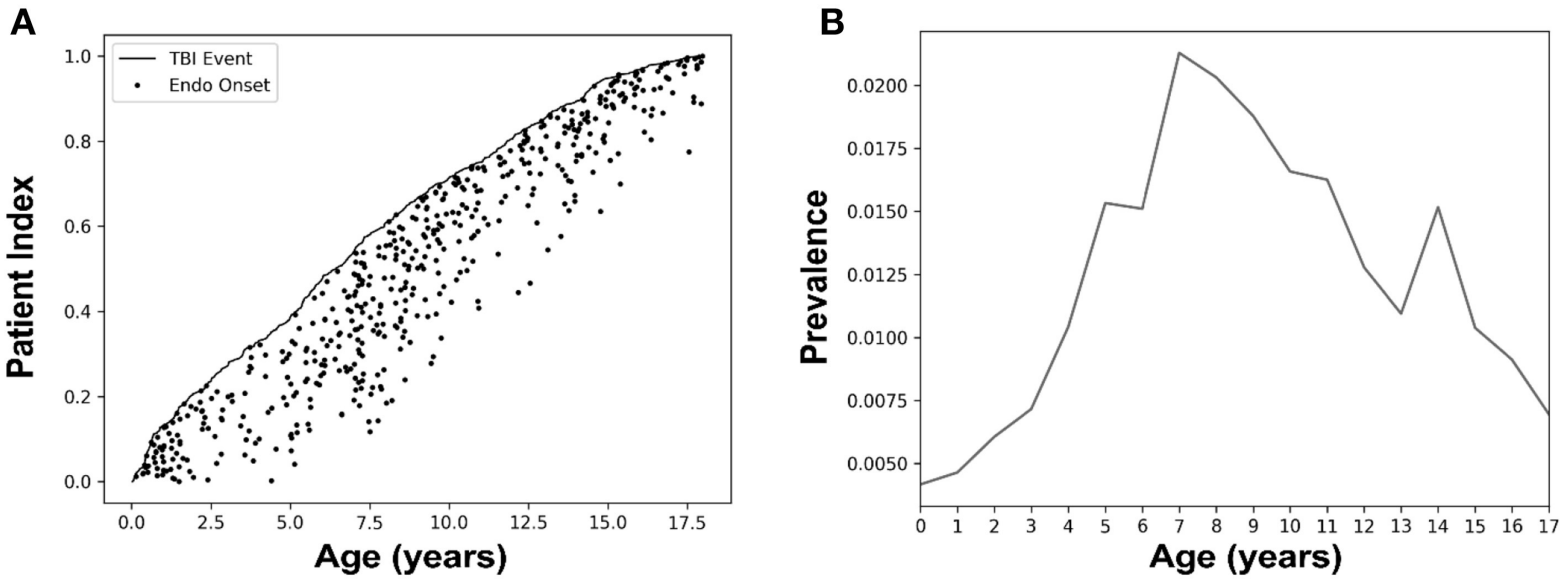
Pediatric patients with a traumatic brain injury (TBI) who were diagnosed with a hypothalamic-pituitary disorder showed the highest prevalence in ages 7–11. **(A)** Patients (*n* = 498) with a TBI and subsequent endocrine diagnosis were indexed by age. Here, we sorted all patients of our group of interest by age with patient index referring to the fraction of the total. The black line represents the age of each patient when they were diagnosed with a TBI, and the dot tracking along the x-axis from the line indicates the subsequent onset of their first endocrine diagnosis. **(B)** Prevalence of an endocrine diagnosis after a TBI stratified by age at the endocrine diagnosis. Here, prevalence was calculated as the number of TBI patients with an endocrine diagnosis divided by those TBI patients without an endocrine. Children aged 7–11 years had the highest prevalence of an endocrine disorder diagnosis after a TBI diagnosis compared to other age groups.

The 498 patients with a TBI and subsequent endocrine diagnosis were indexed by age to show age at TBI and age at endocrine diagnosis (Figure 1A). The 18-year upper age limit for both TBI and endocrine diagnosis sets an arbitrary ceiling effect on the data. The overall prevalence for an endocrine diagnosis after TBI was 0.103 (±0.003 at CI of 90%), with the highest prevalence of an endocrine disorder diagnosis after a TBI diagnosis occurring in the age range of 7–11 years old (Figure 1B). The overall incidence was 0.0014 (±0.0002 at CI of 90%). We calculated a relative risk of 3.22 (±0.29 at CI of 90%), and an odds ratio of 3.24 (±0.29 at CI of 90%), indicating that patients exposed to pediatric TBI had about 3 times the risk of a central endocrine diagnosis compared to the general population. We observe an average number needed to harm of 151.19 (±6.12 at CI of 90%), meaning that for every 151 children diagnosed with a TBI, one would have a hypothalamic-pituitary disorder.

### Male/Female Differences With Age

**Figure 2:**
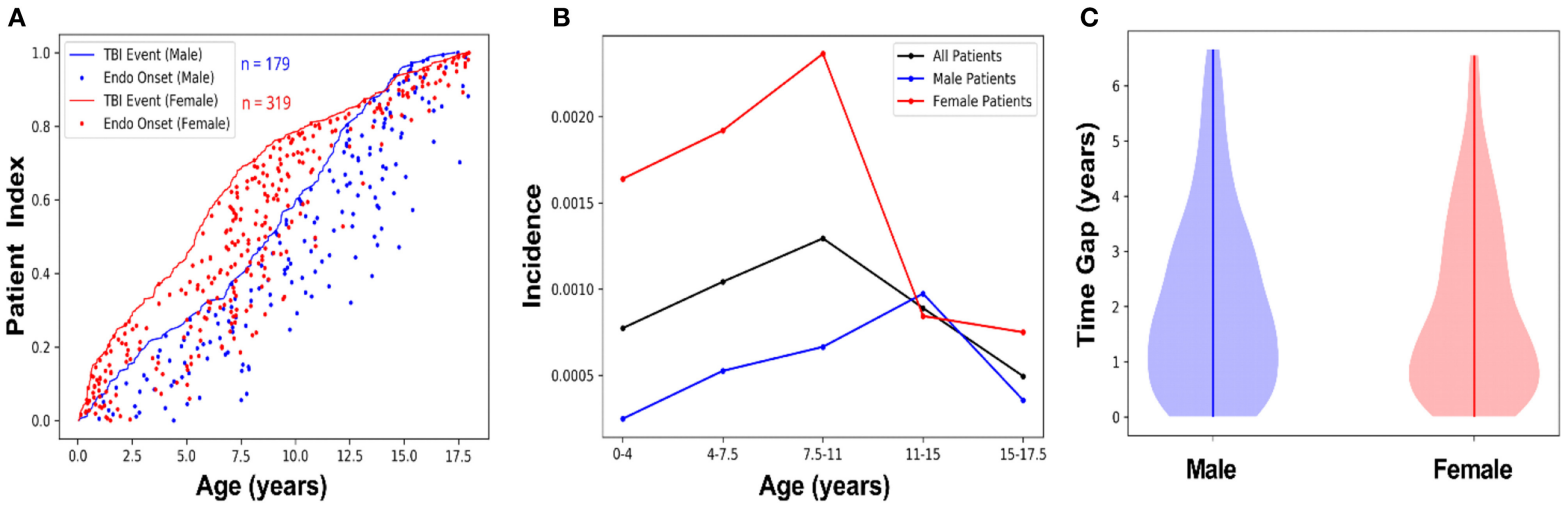
Female subjects show a higher incidence, and an early age of onset, of hypothalamic-pituitary disorder following TBI compared to male subjects. **(A)** Male and female patients with a TBI diagnosis and subsequent endocrine diagnosis were indexed by age, as a fraction of the total number of subjects in each group. The lines represent male (*n* = 179; blue) and female (*n* = 319; red) age of each patient when they were diagnosed with a TBI, and the dots tracking along the x-axis from the line indicate male (blue) and female (red) age at the time of their first endocrine diagnosis. **(B)** Endocrine diagnosis incidence rates after TBI were calculated for male/female patients in blocks of 4-year age groups, with children older than 0 and ≤ 4 years old in the first block, followed by children >4 years old and ≤ 7.5 years old in the second block, etc. Female subjects diagnosed with a TBI (red line) were more likely to have a central endocrine diagnosis at an earlier age compared with male subjects diagnosed with a TBI (blue line). The overall incidence of endocrine dysfunction peaked between ages 7 and 11. This is driven by the higher overall incidence of post-TBI endocrine diagnoses in female patients, compared to male patients, whose incidence peaks between ages 11 and 15. Incidence rate was calculated as the number of new cases per year of endocrine diagnoses in TBI patients divided by the cumulative population of TBI patients. **(C)** Data stratified by male/female indicated female patients showed a shorter time gap between diagnosis of TBI and subsequent endocrine diagnosis compared with male subjects, but not necessarily reflect the onset of undiagnosed symptoms.

The AHCCCS dataset contained binary entries for male and female as entered by the health care facility, which were used to calculate prevalence and incidence. Table 3 shows the prevalence of hypothalamic-pituitary disorder after a TBI diagnosis for male subjects and female subjects across age ranges. In the sample of children with a TBI diagnosis followed by an endocrine diagnosis, female individuals (*n* = 319) outnumbered male individuals (*n* = 179). However, the prevalence of an endocrine diagnosis after a TBI diagnosis in female patients (0.758) was almost three times the prevalence in male patients (0.278; Table 3). The greatest number of TBIs with subsequent endocrine diagnoses in female patients occurred at <2 and 5–8 years of age, whereas male patients had the most between 8 and 12 years of age (Figure 2A). Regardless, both male subjects and female subjects had a high rate of endocrine diagnoses between 7 and 15 years of age (Figure 2A). By 4-year age band, the incidence associated with the age of an endocrine diagnosis differed between female subjects and male subjects, where female subjects showed higher overall incidence and at younger ages than male subjects (Figure 2B). The time gap between TBI diagnosis and endocrine diagnosis differed between male and female patients (Figure 2C). Both male and female patients were weighted toward an endocrine diagnosis within 2 years of TBI, with the bulk of female patients receiving the diagnosis early.

**Table 3:** Prevalence of hypothalamic-pituitary disorder after a TBI diagnosis for male subjects and female subjects and across age ranges.

	**TBI+, Endo+**	
	**Percent (*n*)**	**Prevalence**
Total	100.00% (498)	0.103
Male	35.94% (179)	0.278
Female	64.06% (319)	0.758
**Age range**	**Prevalence**	
0–0.9 years	0.042	
1–1.9 years	0.046	
2–2.9 years	0.061	
3–3.9 years	0.072	
4–4.9 years	0.104	
5–5.9 years	0.153	
6–6.9 years	0.151	
7–7.9 years	0.213	
8–8.9 years	0.203	
9–9.9 years	0.188	
10–10.9 years	0.167	
11–11.9 years	0.163	
12–12.9 years	0.128	
13–13.9 years	0.109	
14–14.9 years	0.152	
15–15.9 years	0.104	
16–16.9 years	0.091	
17–17.9 years	0.069	

### Predominant ICD-9 Codes Used and Diagnoses Over the Years

Table 4 shows the frequency and the number of patients for each of the 14 endocrine-related ICD-9 codes analyzed in the study, and for the 10 most frequent TBI-related ICD-9 codes reported in the database. The predominant TBI diagnosis code identified in the sample was 959.01 (Head Injury: Unspecified) which comprised ~64% of the subjects. Of note, 15% of subjects had concussion (850.X) TBI diagnosis codes. The predominant endocrine disorder code, based on the number of subjects, found in the current study were 259.1 (Precocious sexual development and puberty; not elsewhere classified) which comprised ~59% of the subjects followed by 253.3 (Pituitary dwarfism/GH deficiencies) which comprised 9% of subjects. The substantial number of unspecified head injury diagnostic codes (959.01) prevented a meaningful analysis between TBI and endocrine disorder diagnoses.

**Table 4:** Top 10 reported ICD-9 codes for TBI-related diagnoses, and 14 ICD-9 codes for endocrine related diagnoses.

**ICD-9 code**	**Description**	**Number of patients**	**Frequency (%)**
**TBI-RELATED**			
959.01	Head injury; unspecified	96,421	66.4
850.00	Concussion with no loss of consciousness	10,446	7.2
850.90	Concussion; unspecified	7,850	5.4
850.50	Concussion with loss of consciousness of unspecified duration	3,865	2.7
802.00	Fracture of face bones	3,822	2.6
850.11	Concussion; with loss of consciousness of 30 min or less	3,562	2.5
854.01	Intracranial injury of other and unspecified nature without mention of open intracranial wound; with no loss of consciousness	2,392	1.6
800.01	Closed fracture of vault of skull without mention of intracranial injury; with no loss of consciousness	1,489	1.0
802.80	Closed fracture of other facial bones	1,109	0.8
801.01	Closed fracture of base of skull without mention of intra cranial injury; with no loss of consciousness	943	0.6
**ENDOCRINE-RELATED**			
259.10	Precocious sexual development and puberty; not elsewhere classified	5,334	59.4
253.30	Pituitary dwarfism	856	9.5
259.00	Delay in sexual development and puberty; not elsewhere classified	731	8.1
253.50	Diabetes Insipidus	407	4.5
253.20	Panhypopituitarism	405	4.5
253.10	Other and unspecified anterior pituitary hyperfunction	319	3.6
253.80	Other disorders of the pituitary and other syndromes of diencephalohypophyseal origin	244	2.7
253.40	Other anterior pituitary disorders	177	2.0
253.90	Unspecified disorder of the pituitary gland and its hypothalamic control	172	1.9
253.60	Other disorders of neurohypophysis	147	1.6
256.39	Other ovarian failure	118	1.3
253.70	Latrogenic pituitary disorders	64	0.7
256.31	Premature menopause	5	0.05
628.1	Infertility; female; of pituitary hypothalamic origin	0	0

## Discussion

This is the first study to determine the epidemiology of new-onset central endocrinopathies after TBI in the pediatric population in Arizona. Our analyses indicated an increased risk of a hypothalamic-pituitary disorder for patients with a history of pediatric TBI diagnosis. We observed important male/female differences, where female patients exhibited a higher incidence peaking at an earlier age range compared to male patients, and with female subjects displaying a higher prevalence of endocrine diagnosis after a TBI diagnosis compared to male subjects. Overall, both incidence and prevalence of endocrine diagnosis following a TBI diagnosis peaked between ages 7–11, roughly within 2 years of the initial TBI diagnosis. Additionally, by using data from over 600,000 Arizona pediatric patients per year, we are the first to successfully estimate the epidemiology, relative risk, odds ratio, and number needed to harm of developing a central endocrinopathy after sustaining a TBI in Arizona Medicaid pediatric patients.

### Mechanisms of Endocrine Dysfunction

The exact mechanisms behind endocrine dysfunction following pediatric TBI are unknown, but may be attributed to direct damage to the hypothalamus or pituitary gland. Together, the hypothalamus and pituitary are an integral system in regulating neuronal and hormonal function. The pituitary gland is regulated by a centrally located collection of neurons in the hypothalamus. The hypothalamus secretes precursors and hormones that travel through the hypophyseal portal blood system to act on receptors in the pituitary. The pituitary gland is a major endocrine gland that secretes hormones necessary for normal physiologic functioning such as growth hormone (GH), thyroid stimulating hormone (TSH), adrenocorticotropic hormone (ACTH), prolactin (PRL), luteinizing hormone (LH), follicle stimulating hormone (FSH), anti-diuretic hormone (ADH), and oxytocin. Injury to either the hypothalamus or pituitary gland due to TBI has been shown, in both pre-clinical and clinical studies, to promote endocrine dysfunction due to imbalance in the hormones regulated by these structures (14, 31–35).

Mechanically, the forces of a TBI can selectively damage the hypothalamus and/or the pituitary gland due to their location close to the base of the skull. The pituitary sits at the base of the brain, encapsulated by the hypophyseal fossa, the innermost aperture of the sella turcica, a small cavity within the sphenoid bone of the human skull. It is because of this position in the skull that the pituitary is susceptible to mechanical injury from the impact of blunt force head trauma. Indeed, early studies assessing pituitary damage following TBI found that necrosis in the pituitary gland occurred after injury in patients that had died from TBI (36). More recent studies confirmed that cell death, vascular compromise/hemorrhage, and diffuse axonal injury can result from TBI and lead to damage of the pituitary [reviewed in (37)]. Moreover, a recent study found that individuals who sustained a skull fracture as a result of TBI had the highest rate of pituitary dysfunction after a 1-year follow up (38). As such, the impact forces that occur during a TBI event may directly damage the pituitary or hypothalamus. Without case details on the type and severity of injury, compounded by the majority of TBI diagnosis codes as head injury—unspecified, the relationship between mode of TBI and pituitary or hypothalamus damage cannot be determined. Future population studies with access to imaging findings could uncover this important relationship.

### Prevalence of Endocrine Diagnosis Following a TBI Diagnosis in the Literature

Studies have reported long-term hypopituitarism in the range of 11–69% for TBI survivors, and endocrinopathies have become increasingly recognized over the past couple decades as a consequence of TBI (7, 20, 39). In 2005, the International Consensus Guidelines published screening guidelines for hypopituitarism after TBI in adults, recommending pituitary function screening for all patients with moderate (Glasgow coma score 9–12) to severe TBI (Glasgow coma score 3–8) (20). Although the hypothalamic-pituitary axis regulates normal childhood development, relatively few studies focus on pediatric patients, and no endocrine dysfunction screening guidelines exist for children diagnosed with a TBI. Retrospective and prospective studies report variable rates of hypopituitarism after childhood TBI (18, 19, 21–23). Among retrospective cohort studies that have investigated new-onset endocrine dysfunction after TBI in children (20, 22, 23), our study is unique in that it contains the largest number of patients in the analysis, and it is also the only study to investigate post-TBI endocrine dysfunction in a large state-wide dataset. Regardless, the prevalence of endocrine dysfunction in the first 6 months after TBI [4–86%; (21, 22, 24)], and at 1–5 years after TBI [10–38%; (18, 19, 23, 37)] in children as currently reported in the literature is not instructive due to the large variability in prevalence. The variability in overall prevalence of pituitary dysfunction after TBI may be due to under recognition by caregivers and health-care providers due to protracted, subtle, and non-specific signs, as well as a broad differential diagnosis (40). Also, more than one endocrine pathway may be disrupted after TBI, further confounding the symptomatology and presentation of these patients.

The majority of endocrine dysfunction post-TBI in pediatric patients are GH deficiencies (21–31%), but children can also experience central adrenal deficiency, diabetes insipidus, central hypothyroidism, hypogonadotropic hypogonadism, and elevated prolactin reported between 6 months to a year after TBI (41). It is known that in adults, endocrine dysfunction may present up to 5 years after the initial TBI, suggesting the need for continuous endocrine monitoring of TBI survivors (42, 43). Moreover, recovery of pituitary function can occur in up to 50% of adult patients with major hormonal deficiencies diagnosed at 3 months post-injury (7, 20, 44).

Importantly, unrecognized hypopituitarism can elevate risk for diabetes, delayed or absent puberty, short stature, metabolic syndrome, adrenal insufficiency, and other endocrine dysfunctions, that can significantly affect patients' quality of life. Screening for endocrine deficiencies in susceptible patients and initiating appropriate hormone replacement therapy may prevent these sequelae and improve the prognosis for recovery. For TBI patients, endocrine dysfunction may be prevented, or the prospect for recovery may be improved, through the application of a systematic screen for endocrine dysfunction and the administration of appropriate, well-studied, and well-tolerated hormone replacement therapy. If TBI occurs near period of elevated growth velocity (10–12 years of age for girls; 13–15 years of age for boys), then patients may risk short stature throughout life. Thus, childhood TBI should initiate regular endocrine surveillance, with accurate height and weight measurements, and blood tests and symptom monitoring every 6 months in the first year and yearly thereafter.

Risk factors of hypothalamic-pituitary disorders after TBI are controversial, without definitive relationships between injury factors and hypopituitarism (26, 45, 46). Importantly, TBI need not be severe to lead to endocrine dysfunction, since repeated less severe TBIs disrupt endocrine function (47). In the current study, the dataset lacked indicators of injury severity and a limited number of TBI ICD-9 codes were used, which prevented analysis of a relationship between TBI severity and hypothalamic-pituitary disorder.

Overall, individuals that suffer pediatric and adult TBI may experience hormonal deficits for many years, with a wide range of symptom expression in these patients. Post-TBI endocrinopathies and hypothalamic-pituitary disorders must be considered in the differential diagnosis of any patient with a history of head trauma, regardless of age at injury, mechanism, or severity. Additional large cohort studies, such as ours, are needed to detail the prevalence of endocrine dysfunction following TBI in order to guide clinical decisions.

### Benefits and Limitations of Health Care Terminologies

For this study, we used ICD-9 diagnostic codes associated with TBI and central endocrine disorders to develop inclusion criteria. ICD-9 and Current Procedural Terminology (CPT) billing codes for reimbursement are a vital part of health-care operations (48, 49). These terminologies or codes are generally specific to a particular disease, syndrome, or diagnosis of each patient. These codes are entered into hospital records for every patient visit, regardless of acuity. For example, a patient at her 11th office visit for GH deficiency will have the relevant diagnostic code entered, despite it being the 11th visit for this chronic condition. The physician will also submit a billing code along with the diagnostic code for GH deficiency. Billing codes are coupled with diagnostic codes to help health insurance companies determine the reason for the visit as well as the appropriate reimbursement rate. ICD-9 codes have inherent flaws both with the level of detail and selection of codes by the physician. Selection bias is demonstrated by the predominant TBI ICD-9 code in AHCCCS as 959.01 (Head Injury: unspecified), when there are over 400 other codes to select. It is suspected that the ICD-9 code for “Head Injury: unspecified” and the code for “concussion” may represent a mix of all TBI types which limited our ability in the current study to stratify data and analyze if a specific mechanism of TBI was associated with a higher frequency of central endocrinopathies. Moreover, the use of ICD-9 codes limits interpretation and access to information regarding previous neurological disorders and/or prescribed medications, where steroids and medication for attention deficit hyperactivity disorder may increase risk for endocrine disorders.

Another limitation of our sample is that our data are derived from the Medicaid population of Arizona. Medicaid is the Federal and State program within the United States that subsidizes the cost of medical care for individuals and families with low income and limited resources. In Arizona, the Arizona Health Care Cost Containment Service (AHCCCS) is the Medicaid program, funded by both the Federal and State government, which provides medical insurance coverage for individuals and families. In the year 2011, a half-way point of the years sampled in our study (2008–2014), in order to qualify for this Medicaid program (AHCCCS) individuals were required to meet the following requirements: must be an Arizona resident, must be a United States citizen or qualified immigrant, must have or have applied for a social security number, and must be under the income limit. In 2011, the income limit of a family's income was required to be at or below 133% of the federal poverty level of $22,350/year for a family of four. As such, this sample population represents a unique group of individuals and may not generalize to the entire population. These shortcomings are inherent in any large database, however they are recognized and assumed to be equal among age and male/female patients.

Other limitations of the study include patients who were missed due to the nature of data collection in AHCCCS, due to underreporting of symptoms of endocrine disorders, and due to the large number of patients who do not report TBI. Our data show that most endocrine diagnoses occur in the 2 years following a TBI diagnosis. As such, there is a large cohort of individuals who experience a TBI event from the ages of 15–18 and possibly later go on to develop an endocrine disorder in the next years, but the AHCCCS database does not include children over the age of 18 years. As such, we may have missed patients who suffered a TBI in their late teens and later developed an endocrine disorder over the 18-year-old exclusion criteria. Moreover, some studies report that endocrine disorders during this age range may be underreported or not recognized as a TBI-induced endocrinopathy (50). Additionally, TBI can often go unreported (51, 52), especially when they are mild injuries. However, in the adult literature it has been shown that even mild injuries can lead to dysfunctions of the endocrine system (37, 53). Therefore, these patients may also have been missed in our cohort analysis.

### Significance and Rational for the Research Question

Despite the wide-ranging reports on pediatric TBIs affecting endocrine function, there is little published epidemiological data analyzing large cohorts of patients to identify correlations with male and female patients and age. Additionally, assessing endocrine function is not commonly considered in a clinician's differential diagnosis for a patient presenting to the office after TBI, nor is a history of TBI routinely asked for in a patient presenting with fatigue, weight gain, obesity, delayed puberty, and growth stunting. Our study, and others like it, help emphasize the importance of screening for endocrine dysfunction through diligent history taking and serum endocrine analysis in all patients with a history of pediatric TBI. Moreover, predictive measures are needed in order to better determine which children should undergo further endocrine evaluation after TBI. In order to better care for patients, it is of high importance to develop standard protocols to accurately diagnosis endocrine dysfunction following TBI. Results from this project will help to increase awareness among pediatricians, pediatric endocrinologists, pediatric neurologists, and other clinicians treating children with TBIs, of the incidence and risks of endocrine disorders among TBI survivors. Patients can present with central, new-onset endocrinopathies days to years after TBI, and further assessments of risk-factors and characteristics that contribute to endocrine dysfunction after TBI are critical. Physicians must be aware of endocrine symptoms after TBI and add TBI-induced central endocrinopathies to their differential diagnosis when treating a patient with a history of TBI.

## Data Availability Statement

The datasets generated for this study are available on request to the corresponding author.

## Ethics Statement

The studies involving human participants were reviewed and approved by Phoenix Children's Hospital Institutional Review Board. Written informed consent to participate in this study was provided by the participants' legal guardian/next of kin.

## Author Contributions

AS and JL initiated the worked and developed an experimental plan. GH was consulted and developed the algorithms for data extraction from CHiR with AS and JL. AS, BB, JO, RR, JL, and VS worked to analyze the data, develop the data tables, and figures. BB and VS drafted a technical report to summarize the results, from which AS and JO wrote the manuscript. PA, KL, and OO are consulting physicians with expertise in TBI and/or endocrine disorders and helped frame the research questions, develop the inclusion/exclusion criteria, select ICD-9 codes, and reviewed the manuscript. All authors contributed to the interpretation of results and provided critical feedback on the manuscript prior to submission.

### Conflict of Interest

The authors declare that the research was conducted in the absence of any commercial or financial relationships that could be construed as a potential conflict of interest.
