# Supplementary material for: Epidemiology of Pediatric Traumatic Brain Injury and Hypothalamic-Pituitary Disorders in Arizona
Source: Front Neurol. 2020 Jan 22;10:1410. doi: 10.3389/fneur.2019.01410 (PMC6988738; doi:10.3389/fneur.2019.01410)
Supplement: Supplementary file 1 [file Table_1.DOCX]

Appendix 1: ICD-9 codes for traumatic brain injury diagnosis

**ICD-9 Description**

800.00 Fracture of vault of skull

800.01 Closed fracture of vault of skull without mention of intracranial injury; with no loss of consciousness

800.02 Closed fracture of vault of skull without mention of intracranial injury; with brief [less than one hour] loss of consciousness

800.03 Closed fracture of vault of skull without mention of intracranial injury; with moderate [1-24 hours] loss of consciousness

800.05 Closed fracture of vault of skull without mention of intracranial injury; with prolonged [more than 24 hours] loss of consciousness; without return to pre-existing conscious level

800.06 Closed fracture of vault of skull without mention of intracranial injury; with loss of consciousness of unspecified duration

800.09 Closed fracture of vault of skull without mention of intracranial injury; with concussion; unspecified

800.10 Closed fracture of vault of skull with cerebral laceration and contusion; unspecified state of consciousness

800.11 Closed fracture of vault of skull with cerebral laceration and contusion; with no loss of consciousness

800.12 Closed fracture of vault of skull with cerebral laceration and contusion; with brief [less than one hour] loss of consciousness

800.13 Closed fracture of vault of skull with cerebral laceration and contusion; with moderate [1-24 hours] loss of consciousness

800.14 Closed fracture of vault of skull with cerebral laceration and contusion; with prolonged [more than 24 hours] loss of consciousness and return to pre-existing conscious level

800.15 Closed fracture of vault of skull with cerebral laceration and contusion; with prolonged [more than 24 hours] loss of consciousness; without return to pre-existing conscious level

800.16 Closed fracture of vault of skull with cerebral laceration and contusion; with loss of consciousness of unspecified duration

800.19 Closed fracture of vault of skull with cerebral laceration and contusion; with concussion; unspecified

800.20 Closed fracture of vault of skull with subarachnoid; subdural; and extradural hemorrhage; unspecified state of consciousness

800.21 Closed fracture of vault of skull with subarachnoid; subdural; and extradural hemorrhage; with no loss of consciousness

800.22 Closed fracture of vault of skull with subarachnoid; subdural; and extradural hemorrhage; with brief [less than one hour] loss of consciousness

800.23 Closed fracture of vault of skull with subarachnoid; subdural; and extradural hemorrhage; with moderate [1-24 hours] loss of consciousness

800.24 Closed fracture of vault of skull with subarachnoid; subdural; and extradural hemorrhage; with prolonged [more than 24 hours] loss of consciousness and return to pre-existing conscious level

800.25 Closed fracture of vault of skull with subarachnoid; subdural; and extradural hemorrhage; with prolonged [more than 24 hours] loss of consciousness; without return to pre-existing conscious level

800.26 Closed fracture of vault of skull with subarachnoid; subdural; and extradural hemorrhage; with loss of consciousness of unspecified duration

800.29 Closed fracture of vault of skull with subarachnoid; subdural; and extradural hemorrhage; with concussion; unspecified

800.30 Closed fracture of vault of skull with other and unspecified intracranial hemorrhage; unspecified state of consciousness

800.31 Closed fracture of vault of skull with other and unspecified intracranial hemorrhage; with no loss of consciousness

800.32 Closed fracture of vault of skull with other and unspecified intracranial hemorrhage; with brief [less than one hour] loss of consciousness

800.35 Closed fracture of vault of skull with other and unspecified intracranial hemorrhage; with prolonged [more than 24 hours] loss of consciousness; without return to pre-existing conscious level

800.36 Closed fracture of vault of skull with other and unspecified intracranial hemorrhage; with loss of consciousness of unspecified duration

800.39 Closed fracture of vault of skull with other and unspecified intracranial hemorrhage; with concussion; unspecified

800.40 Closed fracture of vault of skull with intracranial injury of other and unspecified nature; unspecified state of consciousness

800.41 Closed fracture of vault of skull with intracranial injury of other and unspecified nature; with no loss of consciousness

800.42 Closed fracture of vault of skull with intracranial injury of other and unspecified nature; with brief [less than one hour] loss of consciousness

800.45 Closed fracture of vault of skull with intracranial injury of other and unspecified nature; with prolonged [more than 24 hours] loss of consciousness; without return to pre-existing conscious level

800.46 Closed fracture of vault of skull with intracranial injury of other and unspecified nature; with loss of consciousness of unspecified duration

800.49 Closed fracture of vault of skull with intracranial injury of other and unspecified nature; with concussion; unspecified

800.50 Open fracture of vault of skull without mention of intracranial injury; unspecified state of consciousness

800.51 Open fracture of vault of skull without mention of intracranial injury; with no loss of consciousness

800.52 Open fracture of vault of skull without mention of intracranial injury; with brief [less than one hour] loss of consciousness

800.53 Open fracture of vault of skull without mention of intracranial injury; with moderate [1-24 hours] loss of consciousness

800.54 Open fracture of vault of skull without mention of intracranial injury; with prolonged [more than 24 hours] loss of consciousness and return to pre-existing conscious level

800.56 Open fracture of vault of skull without mention of intracranial injury; with loss of consciousness of unspecified duration

800.59 Open fracture of vault of skull without mention of intracranial injury; with concussion; unspecified

800.60 Open fracture of vault of skull with cerebral laceration and contusion; unspecified state of consciousness

800.61 Open fracture of vault of skull with cerebral laceration and contusion; with no loss of consciousness

800.62 Open fracture of vault of skull with cerebral laceration and contusion; with brief [less than one hour] loss of consciousness

800.65 Open fracture of vault of skull with cerebral laceration and contusion; with prolonged [more than 24 hours] loss of consciousness; without return to pre-existing conscious level

800.66 Open fracture of vault of skull with cerebral laceration and contusion; with loss of consciousness of unspecified duration

802.60 Closed fracture of orbital floor (blow-out)

800.70 Open fracture of vault of skull with subarachnoid; subdural; and extradural hemorrhage; unspecified state of consciousness

800.71 Open fracture of vault of skull with subarachnoid; subdural; and extradural hemorrhage; with no loss of consciousness

800.72 Open fracture of vault of skull with subarachnoid; subdural; and extradural hemorrhage; with brief [less than one hour] loss of consciousness

800.74 Open fracture of vault of skull with subarachnoid; subdural; and extradural hemorrhage; with prolonged [more than 24 hours] loss of consciousness and return to pre-existing conscious level

800.75 Open fracture of vault of skull with subarachnoid; subdural; and extradural hemorrhage; with prolonged [more than 24 hours] loss of consciousness; without return to pre-existing conscious level

800.76 Open fracture of vault of skull with subarachnoid; subdural; and extradural hemorrhage; with loss of consciousness of unspecified duration

800.79 Open fracture of vault of skull with subarachnoid; subdural; and extradural hemorrhage; with concussion; unspecified

800.80 Open fracture of vault of skull with other and unspecified intracranial hemorrhage; unspecified state of consciousness

800.81 Open fracture of vault of skull with other and unspecified intracranial hemorrhage; with no loss of consciousness

800.84 Open fracture of vault of skull with other and unspecified intracranial hemorrhage; with prolonged [more than 24 hours] loss of consciousness and return to pre-existing conscious level

800.85 Open fracture of vault of skull with other and unspecified intracranial hemorrhage; with prolonged [more than 24 hours] loss of consciousness; without return to pre-existing conscious level

800.86 Open fracture of vault of skull with other and unspecified intracranial hemorrhage; with loss of consciousness of unspecified duration

800.90 Open fracture of vault of skull with intracranial injury of other and unspecified nature; unspecified state of consciousness

800.91 Open fracture of vault of skull with intracranial injury of other and unspecified nature; with no loss of consciousness

800.93 Open fracture of vault of skull with intracranial injury of other and unspecified nature; with moderate [1-24 hours] loss of consciousness

800.95 Open fracture of vault of skull with intracranial injury of other and unspecified nature; with prolonged [more than 24 hours] loss of consciousness; without return to pre-existing conscious level

800.99 Open fracture of vault of skull with intracranial injury of other and unspecified nature; with concussion; unspecified

801.00 Fracture of base of skull

801.01 Closed fracture of base of skull without mention of intra cranial injury; with no loss of consciousness

801.02 Closed fracture of base of skull without mention of intra cranial injury; with brief [less than one hour] loss of consciousness

801.03 Closed fracture of base of skull without mention of intra cranial injury; with moderate [1-24 hours] loss of consciousness

801.05 Closed fracture of base of skull without mention of intra cranial injury; with prolonged [more than 24 hours] loss of consciousness; without return to pre-existing conscious level

801.06 Closed fracture of base of skull without mention of intra cranial injury; with loss of consciousness of unspecified duration

801.09 Closed fracture of base of skull without mention of intra cranial injury; with concussion; unspecified

801.10 Closed fracture of base of skull with cerebral laceration and contusion; unspecified state of consciousness

801.11 Closed fracture of base of skull with cerebral laceration and contusion; with no loss of consciousness

801.12 Closed fracture of base of skull with cerebral laceration and contusion; with brief [less than one hour] loss of consciousness

801.13 Closed fracture of base of skull with cerebral laceration and contusion; with moderate [1-24 hours] loss of consciousness

801.14 Closed fracture of base of skull with cerebral laceration and contusion; with prolonged [more than 24 hours] loss of consciousness and return to pre-existing conscious level

801.15 Closed fracture of base of skull with cerebral laceration and contusion; with prolonged [more than 24 hours] loss of consciousness; without return to pre-existing conscious level

801.16 Closed fracture of base of skull with cerebral laceration and contusion; with loss of consciousness of unspecified duration

801.19 Closed fracture of base of skull with cerebral laceration and contusion; with concussion; unspecified

801.20 Closed fracture of base of skull with subarachnoid; subdural; and extradural hemorrhage; unspecified state of consciousness

801.21 Closed fracture of base of skull with subarachnoid; subdural; and extradural hemorrhage; with no loss of consciousness

801.22 Closed fracture of base of skull with subarachnoid; subdural; and extradural hemorrhage; with brief [less than one hour] loss of consciousness

801.23 Closed fracture of base of skull with subarachnoid; subdural; and extradural hemorrhage; with moderate [1-24 hours] loss of consciousness

801.24 Closed fracture of base of skull with subarachnoid; subdural; and extradural hemorrhage; with prolonged [more than 24 hours] loss of consciousness and return to pre-existing conscious level

801.25 Closed fracture of base of skull with subarachnoid; subdural; and extradural hemorrhage; with prolonged [more than 24 hours] loss of consciousness; without return to pre-existing conscious level

801.26 Closed fracture of base of skull with subarachnoid; subdural; and extradural hemorrhage; with loss of consciousness of unspecified duration

801.29 Closed fracture of base of skull with subarachnoid; subdural; and extradural hemorrhage; with concussion; unspecified

801.30 Closed fracture of base of skull with other and unspecified intracranial hemorrhage; unspecified state of consciousness

801.31 Closed fracture of base of skull with other and unspecified intracranial hemorrhage; with no loss of consciousness

801.32 Closed fracture of base of skull with other and unspecified intracranial hemorrhage; with brief [less than one hour] loss of consciousness

801.33 Closed fracture of base of skull with other and unspecified intracranial hemorrhage; with moderate [1-24 hours] loss of consciousness

801.34 Closed fracture of base of skull with other and unspecified intracranial hemorrhage; with prolonged [more than 24 hours] loss of consciousness and return to pre-existing conscious level

802.70 Open fracture of orbital floor (blow-out)

802.80 Closed fracture of other facial bones

801.35 Closed fracture of base of skull with other and unspecified intracranial hemorrhage; with prolonged [more than 24 hours] loss of consciousness; without return to pre-existing conscious level

801.36 Closed fracture of base of skull with other and unspecified intracranial hemorrhage; with loss of consciousness of unspecified duration

801.39 Closed fracture of base of skull with other and unspecified intracranial hemorrhage; with concussion; unspecified

801.40 Closed fracture of base of skull with intracranial injury of other and unspecified nature; unspecified state of consciousness

801.41 Closed fracture of base of skull with intracranial injury of other and unspecified nature; with no loss of consciousness

801.42 Closed fracture of base of skull with intracranial injury of other and unspecified nature; with brief [less than one hour] loss of consciousness

801.45 Closed fracture of base of skull with intracranial injury of other and unspecified nature; with prolonged [more than 24 hours] loss of consciousness; without return to pre-existing conscious level

801.46 Closed fracture of base of skull with intracranial injury of other and unspecified nature; with loss of consciousness of unspecified duration

801.49 Closed fracture of base of skull with intracranial injury of other and unspecified nature; with concussion; unspecified

801.50 Open fracture of base of skull without mention of intracranial injury; unspecified state of consciousness

801.51 Open fracture of base of skull without mention of intracranial injury; with no loss of consciousness

801.52 Open fracture of base of skull without mention of intracranial injury; with brief [less than one hour] loss of consciousness

801.56 Open fracture of base of skull without mention of intracranial injury; with loss of consciousness of unspecified duration

801.59 Open fracture of base of skull without mention of intracranial injury; with concussion; unspecified

801.60 Open fracture of base of skull with cerebral laceration and contusion; unspecified state of consciousness

801.61 Open fracture of base of skull with cerebral laceration and contusion; with no loss of consciousness

801.62 Open fracture of base of skull with cerebral laceration and contusion; with brief [less than one hour] loss of consciousness

801.64 Open fracture of base of skull with cerebral laceration and contusion; with prolonged [more than 24 hours] loss of consciousness and return to pre-existing conscious level

801.65 Open fracture of base of skull with cerebral laceration and contusion; with prolonged [more than 24 hours] loss of consciousness; without return to pre-existing conscious level

801.66 Open fracture of base of skull with cerebral laceration and contusion; with loss of consciousness of unspecified duration

801.70 Open fracture of base of skull with subarachnoid; subdural; and extradural hemorrhage; unspecified state of consciousness

801.71 Open fracture of base of skull with subarachnoid; subdural; and extradural hemorrhage; with no loss of consciousness

801.74 Open fracture of base of skull with subarachnoid; subdural; and extradural hemorrhage; with prolonged [more than 24 hours] loss of consciousness and return to pre-existing conscious level

801.75 Open fracture of base of skull with subarachnoid; subdural; and extradural hemorrhage; with prolonged [more than 24 hours] loss of consciousness; without return to pre-existing conscious level

801.76 Open fracture of base of skull with subarachnoid; subdural; and extradural hemorrhage; with loss of consciousness of unspecified duration

801.80 Open fracture of base of skull with other and unspecified intracranial hemorrhage; unspecified state of consciousness

801.81 Open fracture of base of skull with other and unspecified intracranial hemorrhage; with no loss of consciousness

801.84 Open fracture of base of skull with other and unspecified intracranial hemorrhage; with prolonged [more than 24 hours] loss of consciousness and return to pre-existing conscious level

801.85 Open fracture of base of skull with other and unspecified intracranial hemorrhage; with prolonged [more than 24 hours] loss of consciousness; without return to pre-existing conscious level

801.86 Open fracture of base of skull with other and unspecified intracranial hemorrhage; with loss of consciousness of unspecified duration

801.90 Open fracture of base of skull with intracranial injury of other and unspecified nature; unspecified state of consciousness

801.91 Open fracture of base of skull with intracranial injury of other and unspecified nature; with no loss of consciousness

801.92 Open fracture of base of skull with intracranial injury of other and unspecified nature; with brief [less than one hour] loss of consciousness

801.95 Open fracture of base of skull with intracranial injury of other and unspecified nature; with prolonged [more than 24 hours] loss of consciousness; without return to pre-existing conscious level

801.96 Open fracture of base of skull with intracranial injury of other and unspecified nature; with loss of consciousness of unspecified duration

802.00 Fracture of face bones

802.10 Open fracture of nasal bones

802.40 Closed fracture of malar and maxillary bones

802.50 Open fracture of malar and maxillary bones

802.90 Open fracture of other facial bones

803.00 Other and unqualified skull fractures

803.01 Other closed skull fracture without mention of intracranial injury; with no loss of consciousness

803.02 Other closed skull fracture without mention of intracranial injury; with brief [less than one hour] loss of consciousness

803.03 Other closed skull fracture without mention of intracranial injury; with moderate [1-24 hours] loss of consciousness

803.04 Other closed skull fracture without mention of intracranial injury; with prolonged [more than 24 hours] loss of consciousness and return to pre-existing conscious level

803.05 Other closed skull fracture without mention of intracranial injury; with prolonged [more than 24 hours] loss of consciousness; without return to pre-existing conscious level

803.06 Other closed skull fracture without mention of intracranial injury; with loss of consciousness of unspecified duration

803.09 Other closed skull fracture without mention of intracranial injury; with concussion; unspecified

803.10 Other closed skull fracture with cerebral laceration and contusion; unspecified state of consciousness

803.11 Other closed skull fracture with cerebral laceration and contusion; with no loss of consciousness

803.12 Other closed skull fracture with cerebral laceration and contusion; with brief [less than one hour] loss of consciousness

803.13 Other closed skull fracture with cerebral laceration and contusion; with moderate [1-24 hours] loss of consciousness

803.14 Other closed skull fracture with cerebral laceration and contusion; with prolonged [more than 24 hours] loss of consciousness and return to pre-existing conscious level

803.15 Other closed skull fracture with cerebral laceration and contusion; with prolonged [more than 24 hours] loss of consciousness; without return to pre-existing conscious level

803.16 Other closed skull fracture with cerebral laceration and contusion; with loss of consciousness of unspecified duration

803.19 Other closed skull fracture with cerebral laceration and contusion; with concussion; unspecified

803.20 Other closed skull fracture with subarachnoid; subdural; and extradural hemorrhage; unspecified state of consciousness

803.21 Other closed skull fracture with subarachnoid; subdural; and extradural hemorrhage; with no loss of consciousness

803.22 Other closed skull fracture with subarachnoid; subdural; and extradural hemorrhage; with brief [less than one hour] loss of consciousness

803.23 Other closed skull fracture with subarachnoid; subdural; and extradural hemorrhage; with moderate [1-24 hours] loss of consciousness

803.24 Other closed skull fracture with subarachnoid; subdural; and extradural hemorrhage; with prolonged [more than 24 hours] loss of consciousness and return to pre-existing conscious level

803.25 Other closed skull fracture with subarachnoid; subdural; and extradural hemorrhage; with prolonged [more than 24 hours] loss of consciousness; without return to pre-existing conscious level

803.26 Other closed skull fracture with subarachnoid; subdural; and extradural hemorrhage; with loss of consciousness of unspecified duration

803.29 Other closed skull fracture with subarachnoid; subdural; and extradural hemorrhage; with concussion; unspecified

803.30 Other closed skull fracture with other and unspecified intracranial hemorrhage; unspecified state of unconsciousness

803.31 Other closed skull fracture with other and unspecified intracranial hemorrhage; with no loss of consciousness

803.32 Other closed skull fracture with other and unspecified intracranial hemorrhage; with brief [less than one hour] loss of consciousness

803.33 Other closed skull fracture with other and unspecified intracranial hemorrhage; with moderate [1-24 hours] loss of consciousness

803.34 Other closed skull fracture with other and unspecified intracranial hemorrhage; with prolonged [more than 24 hours] loss of consciousness and return to pre-existing conscious level

803.35 Other closed skull fracture with other and unspecified intracranial hemorrhage; with prolonged [more than 24 hours] loss of consciousness; without return to pre-existing conscious level

803.36 Other closed skull fracture with other and unspecified intracranial hemorrhage; with loss of consciousness of unspecified duration

803.39 Other closed skull fracture with other and unspecified intracranial hemorrhage; with concussion; unspecified

803.40 Other closed skull fracture with intracranial injury of other and unspecified nature; unspecified state of consciousness

803.41 Other closed skull fracture with intracranial injury of other and unspecified nature; with no loss of consciousness

803.42 Other closed skull fracture with intracranial injury of other and unspecified nature; with brief [less than one hour] loss of consciousness

803.43 Other closed skull fracture with intracranial injury of other and unspecified nature; with moderate [1-24 hours] loss of consciousness

803.44 Other closed skull fracture with intracranial injury of other and unspecified nature; with prolonged [more than 24 hours] loss of consciousness and return to pre-existing conscious level

803.45 Other closed skull fracture with intracranial injury of other and unspecified nature; with prolonged [more than 24 hours] loss of consciousness; without return to pre-existing conscious level

803.46 Other closed skull fracture with intracranial injury of other and unspecified nature; with loss of consciousness of unspecified duration

803.49 Other closed skull fracture with intracranial injury of other and unspecified nature; with concussion; unspecified

803.50 Other open skull fracture without mention of injury; unspecified state of consciousness

803.51 Other open skull fracture without mention of intracranial injury; with no loss of consciousness

803.52 Other open skull fracture without mention of intracranial injury; with brief [less than one hour] loss of consciousness

803.55 Other open skull fracture without mention of intracranial injury; with prolonged [more than 24 hours] loss of consciousness; without return to pre-existing conscious level

803.56 Other open skull fracture without mention of intracranial injury; with loss of consciousness of unspecified duration

803.59 Other open skull fracture without mention of intracranial injury; with concussion; unspecified

803.60 Other open skull fracture with cerebral laceration and contusion; unspecified state of consciousness

803.61 Other open skull fracture with cerebral laceration and contusion; with no loss of consciousness

803.65 Other open skull fracture with cerebral laceration and contusion; with prolonged [more than 24 hours] loss of consciousness; without return to pre-existing conscious level

803.66 Other open skull fracture with cerebral laceration and contusion; with loss of consciousness of unspecified duration

803.69 Other open skull fracture with cerebral laceration and contusion; with concussion; unspecified

803.70 Other open skull fracture with subarachnoid; subdural; and extradural hemorrhage; unspecified state of consciousness

803.71 Other open skull fracture with subarachnoid; subdural; and extradural hemorrhage; with no loss of consciousness

803.72 Other open skull fracture with subarachnoid; subdural; and extradural hemorrhage; with brief [less than one hour] loss of consciousness

803.74 Other open skull fracture with subarachnoid; subdural; and extradural hemorrhage; with prolonged [more than 24 hours] loss of consciousness and return to pre-existing conscious level

803.75 Other open skull fracture with subarachnoid; subdural; and extradural hemorrhage; with prolonged [more than 24 hours] loss of consciousness; without return to pre-existing conscious level

803.76 Other open skull fracture with subarachnoid; subdural; and extradural hemorrhage; with loss of consciousness of unspecified duration

803.80 Other open skull fracture with other and unspecified intracranial hemorrhage; unspecified state of consciousness

803.82 Other open skull fracture with other and unspecified intracranial hemorrhage; with brief [less than one hour] loss of consciousness

803.85 Other open skull fracture with other and unspecified intracranial hemorrhage; with prolonged [more than 24 hours] loss of consciousness; without return to pre-existing conscious level

803.86 Other open skull fracture with other and unspecified intracranial hemorrhage; with loss of consciousness of unspecified duration

803.90 Other open skull fracture with intracranial injury of other and unspecified nature; unspecified state of consciousness

803.91 Other open skull fracture with intracranial injury of other and unspecified nature; with no loss of consciousness

803.95 Other open skull fracture with intracranial injury of other and unspecified nature; with prolonged [more than 24 hours] loss of consciousness; without return to pre-existing conscious level

803.96 Other open skull fracture with intracranial injury of other and unspecified nature; with loss of consciousness of unspecified duration

803.99 Other open skull fracture with intracranial injury of other and unspecified nature; with concussion; unspecified

804.00 Multiple fractures involving skull or face with other bones

804.01 Closed fractures involving skull or face with other bones; without mention of intracranial injury; with no loss of consciousness

804.02 Closed fractures involving skull or face with other bones; without mention of intracranial injury; with brief [less than one hour] loss of consciousness

804.04 Closed fractures involving skull or face with other bones; without mention or intracranial injury; with prolonged [more than 24 hours] loss of consciousness and return to pre-existing conscious level

804.05 Closed fractures involving skull of face with other bones; without mention of intracranial injury; with prolonged [more than 24 hours] loss of consciousness; without return to pre-existing conscious level

804.06 Closed fractures involving skull of face with other bones; without mention of intracranial injury; with loss of consciousness of unspecified duration

804.09 Closed fractures involving skull of face with other bones; without mention of intracranial injury; with concussion; unspecified

804.10 Closed fractures involving skull or face with other bones; with cerebral laceration and contusion; unspecified state of consciousness

804.11 Closed fractures involving skull or face with other bones; with cerebral laceration and contusion; with no loss of consciousness

804.13 Closed fractures involving skull or face with other bones; with cerebral laceration and contusion; with moderate [1-24 hours] loss of consciousness

804.14 Closed fractures involving skull or face with other bones; with cerebral laceration and contusion; with prolonged [more than 24 hours] loss of consciousness and return to pre-existing conscious level

804.16 Closed fractures involving skull or face with other bones; with cerebral laceration and contusion; with loss of consciousness of unspecified duration

804.19 Closed fractures involving skull or face with other bones; with cerebral laceration and contusion; with concussion; unspecified

804.20 Closed fractures involving skull or face with other bones with subarachnoid; subdural; and extradural hemorrhage; unspecified state of consciousness

804.21 Closed fractures involving skull or face with other bones with subarachnoid; subdural; and extradural hemorrhage; with no loss of consciousness

804.22 Closed fractures involving skull or face with other bones with subarachnoid; subdural; and extradural hemorrhage; with brief [less than one hour] loss of consciousness

804.23 Closed fractures involving skull or face with other bones with subarachnoid; subdural; and extradural hemorrhage; with moderate [1-24 hours] loss of consciousness

804.24 Closed fractures involving skull or face with other bones with subarachnoid; subdural; and extradural hemorrhage; with prolonged [more than 24 hours] loss of consciousness and return to pre-existing conscious level

804.25 Closed fractures involving skull or face with other bones with subarachnoid; subdural; and extradural hemorrhage; with prolonged [more than 24 hours] loss of consciousness; without return to pre-existing conscious level

804.26 Closed fractures involving skull or face with other bones with subarachnoid; subdural; and extradural hemorrhage; with loss of consciousness of unspecified duration

804.30 Closed fractures involving skull or face with other bones; with other and unspecified intracranial hemorrhage; unspecified state of consciousness

804.31 Closed fractures involving skull or face with other bones; with other and unspecified intracranial hemorrhage; with no loss of consciousness

804.34 Closed fractures involving skull or face with other bones; with other and unspecified intracranial hemorrhage; with prolonged [more than 24 hours] loss of consciousness and return to pre- existing conscious level

804.36 Closed fractures involving skull or face with other bones; with other and unspecified intracranial hemorrhage; with loss of consciousness of unspecified duration

804.39 Closed fractures involving skull or face with other bones; with other and unspecified intracranial hemorrhage; with concussion; unspecified

804.40 Closed fractures involving skull or face with other bones; with intracranial injury of other and unspecified nature; unspecified state of consciousness

804.41 Closed fractures involving skull or face with other bones; with intracranial injury of other and unspecified nature; with no loss of consciousness

804.45 Closed fractures involving skull or face with other bones; with intracranial injury of other and unspecified nature; with prolonged [more than 24 hours] loss of consciousness; without return to pre-existing conscious level

804.46 Closed fractures involving skull or face with other bones; with intracranial injury of other and unspecified nature; with loss of consciousness of unspecified duration

804.50 Open fractures involving skull or face with other bones; without mention of intracranial injury; unspecified state of consciousness

804.52 Open fractures involving skull or face with other bones; without mention of intracranial injury; with brief [less than one hour] loss of consciousness

804.60 Open fractures involving skull or face with other bones; with cerebral laceration and contusion; unspecified state of consciousness

804.61 Open fractures involving skull or face with other bones; with cerebral laceration and contusion; with no loss of consciousness

804.70 Open fractures involving skull or face with other bones with subarachnoid; subdural; and extradural hemorrhage; unspecified state of consciousness

804.71 Open fractures involving skull or face with other bones with subarachnoid; subdural; and extradural hemorrhage; with no loss of consciousness

804.74 Open fractures involving skull or face with other bones with subarachnoid; subdural; and extradural hemorrhage; with prolonged [more than 24 hours] loss of consciousness and return to pre-existing conscious level

804.76 Open fractures involving skull or face with other bones with subarachnoid; subdural; and extradural hemorrhage; with loss of consciousness of unspecified duration

804.80 Open fractures involving skull or face with other bones; with other and unspecified intracranial hemorrhage; unspecified state of consciousness

804.82 Open fractures involving skull or face with other bones; with other and unspecified intracranial hemorrhage; with brief [less than one hour] loss of consciousness

804.89 Open fractures involving skull or face with other bones; with other and unspecified intracranial hemorrhage; with concussion; unspecified

804.90 Open fractures involving skull or face with other bones; with intracranial injury of other and unspecified nature; unspecified state of consciousness

804.96 Open fractures involving skull or face with other bones; with intracranial injury of other and unspecified nature; with loss of consciousness of unspecified duration

804.99 Open fractures involving skull or face with other bones; with intracranial injury of other and unspecified nature; with concussion; unspecified

850.00 Concussion with no loss of consciousness

850.10 Concussion; with brief loss of consciousness

850.11 Concussion; with loss of consciousness of 30 minutes or less

850.12 Concussion; with loss of consciousness from 31 to 59 minutes

850.20 Concussion with moderate loss of consciousness

850.30 Concussion with prolonged loss of consciousness and return to pre-existing conscious level

850.40 Concussion with prolonged loss of consciousness; without return to pre-existing conscious level

850.50 Concussion with loss of consciousness of unspecified duration

850.90 Concussion; unspecified

851.00 Cerebral laceration and contusion

851.01 Cortex (cerebral) contusion without mention of open intracranial wound; with no loss of consciousness

851.02 Cortex (cerebral) contusion without mention of open intracranial wound; with brief [less than one hour] loss of consciousness

851.03 Cortex (cerebral) contusion without mention of open intracranial wound; with moderate [1-24 hours] loss of consciousness

851.04 Cortex (cerebral) contusion without mention of open intracranial wound; with prolonged [more than 24 hours] loss of consciousness and return to pre-existing conscious level

851.05 Cortex (cerebral) contusion without mention of open intracranial wound; with prolonged [more than 24 hours] loss of consciousness without return to pre-existing conscious level

851.06 Cortex (cerebral) contusion without mention of open intracranial wound; with loss of consciousness of unspecified duration

851.09 Cortex (cerebral) contusion without mention of open intracranial wound; with concussion; unspecified

851.10 Cortex (cerebral) contusion with open intracranial wound; unspecified state of consciousness

851.11 Cortex (cerebral) contusion with open intracranial wound; with no loss of consciousness

851.13 Cortex (cerebral) contusion with open intracranial wound; with moderate [1-24 hours] loss of consciousness

851.16 Cortex (cerebral) contusion with open intracranial wound; with loss of consciousness of unspecified duration

851.19 Cortex (cerebral) contusion with open intracranial wound; with concussion; unspecified

851.20 Cortex (cerebral) laceration without mention of open intracranial wound; unspecified state of consciousness

851.21 Cortex (cerebral) laceration without mention of open intracranial wound; with no loss of consciousness

851.22 Cortex (cerebral) laceration without mention of open intracranial wound; with brief [less than one hour] loss of consciousness

851.24 Cortex (cerebral) laceration without mention of open intracranial wound; with prolonged [more than 24 hours] loss of consciousness and return to pre-existing conscious level

851.26 Cortex (cerebral) laceration without mention of open intracranial wound; with loss of consciousness of unspecified duration

851.30 Cortex (cerebral) laceration with open intracranial wound; unspecified state of consciousness

851.31 Cortex (cerebral) laceration with open intracranial wound; with no loss of consciousness

851.32 Cortex (cerebral) laceration with open intracranial wound; with brief [less than one hour] loss of consciousness

851.35 Cortex (cerebral) laceration with open intracranial wound; with prolonged [more than 24 hours] loss of consciousness without return to pre-existing conscious level

851.36 Cortex (cerebral) laceration with open intracranial wound; with loss of consciousness of unspecified duration

851.40 Cerebellar or brain stem contusion without mention of open intracranial wound; unspecified state of consciousness

851.41 Cerebellar or brain stem contusion without mention of open intracranial wound; with no loss of consciousness

851.42 Cerebellar or brain stem contusion without mention of open intracranial wound; with brief [less than one hour] loss of consciousness

851.44 Cerebellar or brain stem contusion without mention of open intracranial wound; with prolonged [more than 24 hours] loss consciousness and return to pre-existing conscious level

851.45 Cerebellar or brain stem contusion without mention of open intracranial wound; with prolonged [more than 24 hours] loss of consciousness without return to pre-existing conscious level

851.46 Cerebellar or brain stem contusion without mention of open intracranial wound; with loss of consciousness of unspecified duration

851.49 Cerebellar or brain stem contusion without mention of open intracranial wound; with concussion; unspecified

851.50 Cerebellar or brain stem contusion with open intracranial wound; unspecified state of consciousness

851.55 Cerebellar or brain stem contusion with open intracranial wound; with prolonged [more than 24 hours] loss of consciousness without return to pre-existing conscious level

851.60 Cerebellar or brain stem laceration without mention of open intracranial wound; unspecified state of consciousness

851.65 Cerebellar or brain stem laceration without mention of open intracranial wound; with prolonged [more than 24 hours] loss of consciousness without return to pre-existing conscious level

851.66 Cerebellar or brain stem laceration without mention of open intracranial wound; with loss of consciousness of unspecified duration

851.70 Cerebellar or brain stem laceration with open intracranial wound; unspecified state of consciousness

851.71 Cerebellar or brain stem laceration with open intracranial wound; with no loss of consciousness

851.75 Cerebellar or brain stem laceration with open intracranial wound; with prolonged [more than 24 hours] loss of consciousness without return to pre-existing conscious level

851.80 Other and unspecified cerebral laceration and contusion; without mention of open intracranial wound; unspecified state of consciousness

851.81 Other and unspecified cerebral laceration and contusion; without mention of open intracranial wound; with no loss of consciousness

851.82 Other and unspecified cerebral laceration and contusion; without mention of open intracranial wound; with brief [less than one hour] loss of consciousness

851.83 Other and unspecified cerebral laceration and contusion; without mention of open intracranial wound; with moderate [1-24 hours] loss of consciousness

851.84 Other and unspecified cerebral laceration and contusion; without mention of open intracranial wound; with prolonged [more than 24 hours] loss of consciousness and return to pre- existing conscious level

851.85 Other and unspecified cerebral laceration and contusion; without mention of open intracranial wound; with prolonged [more than 24 hours] loss of consciousness without return to pre-existing conscious level

851.86 Other and unspecified cerebral laceration and contusion; without mention of open intracranial wound; with loss of consciousness of unspecified duration

851.89 Other and unspecified cerebral laceration and contusion; without mention of open intracranial wound; with concussion; unspecified

851.90 Other and unspecified cerebral laceration and contusion; with open intracranial wound; unspecified state of consciousness

851.91 Other and unspecified cerebral laceration and contusion; with open intracranial wound; with no loss of consciousness

851.92 Other and unspecified cerebral laceration and contusion; with open intracranial wound; with brief [less than one hour] loss of consciousness

851.94 Other and unspecified cerebral laceration and contusion; with open intracranial wound; with prolonged [more than 24 hours] loss of consciousness and return to pre-existing conscious level

851.95 Other and unspecified cerebral laceration and contusion; with open intracranial wound; with prolonged [more than 24 hours] loss of consciousness without return to pre-existing conscious level

851.96 Other and unspecified cerebral laceration and contusion; with open intracranial wound; with loss of consciousness of unspecified duration

851.99 Other and unspecified cerebral laceration and contusion; with open intracranial wound; with concussion; unspecified

852.00 Subarachnoid; subdural; and extradural hemorrhage; following injury

852.01 Subarachnoid hemorrhage following injury without mention of open intracranial wound; with no loss of consciousness

852.02 Subarachnoid hemorrhage following injury without mention of open intracranial wound; with brief [less than one hour] loss of consciousness

852.03 Subarachnoid hemorrhage following injury without mention of open intracranial wound; with moderate [1-24 hours] loss of consciousness

852.04 Subarachnoid hemorrhage following injury without mention of open intracranial wound; with prolonged [more than 24 hours] loss of consciousness and return to pre-existing conscious level

852.05 Subarachnoid hemorrhage following injury without mention of open intracranial wound; with prolonged [more than 24 hours] loss of consciousness without return to pre-existing conscious level

852.06 Subarachnoid hemorrhage following injury without mention of open intracranial wound; with loss of consciousness of unspecified duration

852.09 Subarachnoid hemorrhage following injury without mention of open intracranial wound; with concussion; unspecified

852.10 Subarachnoid hemorrhage following injury with open intracranial wound; unspecified state of consciousness

852.11 Subarachnoid hemorrhage following injury with open intracranial wound; with no loss of consciousness

852.13 Subarachnoid hemorrhage following injury with open intracranial wound; with moderate [1-24 hours] loss of consciousness

852.15 Subarachnoid hemorrhage following injury with open intracranial wound; with prolonged [more than 24 hours] loss of consciousness without return to pre-existing conscious level

852.16 Subarachnoid hemorrhage following injury with open intracranial wound; with loss of consciousness of unspecified duration

852.19 Subarachnoid hemorrhage following injury with open intracranial wound; with concussion; unspecified

852.20 Subdural hemorrhage following injury without mention of open intracranial wound; unspecified state of consciousness

852.21 Subdural hemorrhage following injury without mention of open intracranial wound; with no loss of consciousness

852.22 Subdural hemorrhage following injury without mention of open intracranial wound; with brief [less than one hour] loss of consciousness

852.23 Subdural hemorrhage following injury without mention of open intracranial wound; with moderate [1-24 hours] loss of consciousness

852.24 Subdural hemorrhage following injury without mention of open intracranial wound; with prolonged [more than 24 hours] loss of consciousness and return to pre-existing conscious level

852.25 Subdural hemorrhage following injury without mention of open intracranial wound; with prolonged [more than 24 hours] loss of consciousness without return to pre-existing conscious level

852.26 Subdural hemorrhage following injury without mention of open intracranial wound; with loss of consciousness of unspecified duration

852.29 Subdural hemorrhage following injury without mention of open intracranial wound; with concussion; unspecified

852.30 Subdural hemorrhage following injury with open intracranial wound; unspecified state of consciousness

852.31 Subdural hemorrhage following injury with open intracranial wound; with no loss of consciousness

852.32 Subdural hemorrhage following injury with open intracranial wound; with brief [less than one hour] loss of consciousness

852.33 Subdural hemorrhage following injury with open intracranial wound; with moderate [1-24 hours] loss of consciousness

852.35 Subdural hemorrhage following injury with open intracranial wound; with prolonged [more than 24 hours] loss of consciousness without return to pre-existing conscious level

852.36 Subdural hemorrhage following injury with open intracranial wound; with loss of consciousness of unspecified duration

852.39 Subdural hemorrhage following injury with open intracranial wound; with concussion; unspecified

852.40 Extradural hemorrhage following injury without mention of open intracranial wound; unspecified state of consciousness

852.41 Extradural hemorrhage following injury without mention of open intracranial wound; with no loss of consciousness

852.42 Extradural hemorrhage following injury without mention of open intracranial wound; with brief [less than 1 hour] loss of consciousness

852.43 Extradural hemorrhage following injury without mention of open intracranial wound; with moderate [1-24 hours] loss of consciousness

852.44 Extradural hemorrhage following injury without mention of open intracranial wound; with prolonged [more than 24 hours] loss of consciousness and return to pre-existing conscious level

852.45 Extradural hemorrhage following injury without mention of open intracranial wound; with prolonged [more than 24 hours] loss of consciousness without return to pre-existing conscious level

852.46 Extradural hemorrhage following injury without mention of open intracranial wound; with loss of consciousness of unspecified duration

852.49 Extradural hemorrhage following injury without mention of open intracranial wound; with concussion; unspecified

852.50 Extradural hemorrhage following injury with open intracranial wound; unspecified state of consciousness

852.51 Extradural hemorrhage following injury with open intracranial wound; with no loss of consciousness

852.52 Extradural hemorrhage following injury with open intracranial wound; with brief [less than one hour] loss of consciousness

852.56 Extradural hemorrhage following injury with open intracranial wound; with loss of consciousness of unspecified duration

852.59 Extradural hemorrhage following injury with open intracranial wound; with concussion; unspecified

853.00 Other and unspecified intracranial hemorrhage following injury

853.01 Other and unspecified intracranial hemorrhage following injury without mention of open intracranial wound; with no loss of consciousness

853.02 Other and unspecified intracranial hemorrhage following injury without mention of open intracranial wound; with brief [less than one hour] loss of consciousness

853.03 Other and unspecified intracranial hemorrhage following injury without mention of open intracranial wound; with moderate [1-24 hours] loss of consciousness

853.04 Other and unspecified intracranial hemorrhage following injury without mention of open intracranial wound; with prolonged [more than 24 hours] loss of consciousness and return to pre- existing conscious level

853.05 Other and unspecified intracranial hemorrhage following injury without mention of open intracranial wound; with prolonged [more than 24 hours] loss of consciousness without return to pre-existing conscious level

853.06 Other and unspecified intracranial hemorrhage following injury without mention of open intracranial wound; with loss of consciousness of unspecified duration

853.09 Other and unspecified intracranial hemorrhage following injury without mention of open intracranial wound; with concussion; unspecified

853.10 Other and unspecified intracranial hemorrhage following injury with open intracranial wound; unspecified state of consciousness

853.14 Other and unspecified intracranial hemorrhage following injury with open intracranial wound; with prolonged [more than 24 hours] loss of consciousness and return to pre-existing conscious level

853.15 Other and unspecified intracranial hemorrhage following injury with open intracranial wound; with prolonged [more than 24 hours] loss of consciousness without return to pre-existing conscious level

853.16 Other and unspecified intracranial hemorrhage following injury with open intracranial wound; with loss of consciousness of unspecified duration

853.19 Other and unspecified intracranial hemorrhage following injury with open intracranial wound; with concussion; unspecified

854.00 Intracranial injury of other and unspecified nature without mention of open intracranial wound; unspecified state of consciousness

854.01 Intracranial injury of other and unspecified nature without mention of open intracranial wound; with no loss of consciousness

854.02 Intracranial injury of other and unspecified nature without mention of open intracranial wound; with brief [less than one hour] loss of consciousness

854.03 Intracranial injury of other and unspecified nature without mention of open intracranial wound; with moderate [1-24 hours] loss of consciousness

854.04 Intracranial injury of other and unspecified nature without mention of open intracranial wound; with prolonged [more than 24 hours] loss of consciousness and return to pre-existing conscious level

854.05 Intracranial injury of other and unspecified nature without mention of open intracranial wound; with prolonged [more than 24 hours] loss of consciousness without return to pre-existing conscious level

854.06 Intracranial injury of other and unspecified nature without mention of open intracranial wound; with loss of consciousness of unspecified duration

854.09 Intracranial injury of other and unspecified nature without mention of open intracranial wound; with concussion; unspecified

854.10 Intracranial injury of other and unspecified nature with open intracranial wound; unspecified state of consciousness

854.11 Intracranial injury of other and unspecified nature with open intracranial wound; with no loss of consciousness

854.12 Intracranial injury of other and unspecified nature with open intracranial wound; with brief [less than one hour] loss of consciousness

854.13 Intracranial injury of other and unspecified nature with open intracranial wound; with moderate [1-24 hours] loss of consciousness

854.14 Intracranial injury of other and unspecified nature with open intracranial wound; with prolonged [more than 24 hours] loss of consciousness and return to pre-existing conscious level

854.15 Intracranial injury of other and unspecified nature with open intracranial wound; with prolonged [more than 24 hours] loss of consciousness without return to pre-existing conscious level

854.16 Intracranial injury of other and unspecified nature with open intracranial wound; with loss of consciousness of unspecified duration

854.19 Intracranial injury of other and unspecified nature with open intracranial wound; with concussion; unspecified

925.10 Crushing injury of face and scalp

953.00 Injury to cervical nerve root

959.01 Head injury; unspecified

995.50 Child abuse; unspecified

995.55 Shaken baby syndrome

998.12 Accidental puncture or laceration during a procedure; not elsewhere classified

998.20 Accidental puncture or laceration during a procedure; not elsewhere classified

Appendix 2: ICD-9 codes for central endocrine diagnosis

**ICD-9 Description**

253.10 Other and unspecified anterior pituitary hyperfunction

253.20 Panhypopituitarism

253.30 Pituitary dwarfism

253.40 Other anterior pituitary disorders

253.50 Diabetes insipidus

253.60 Other disorders of neurohypophysis

253.70 Iatrogenic pituitary disorders

253.80 Other disorders of the pituitary and other syndromes of diencephalohypophyseal origin

253.90 Unspecified disorder of the pituitary gland and its hypothalamic control

256.31 Premature menopause

256.39 Other ovarian failure

259.00 Delay in sexual development and puberty; not elsewhere classified

259.10 Precocious sexual development and puberty; not elsewhere classified

628.10 Infertility; female; of pituitary-hypothalamic origin
